# The Bangla clubfoot tool: a repeatability study

**DOI:** 10.1186/1757-1146-7-27

**Published:** 2014-05-06

**Authors:** Angela Margaret Evans, Roksana Perveen, Vikki A Ford-Powell, Simon Barker

**Affiliations:** 1 Department of Podiatry, Lower Extremity and Gait Studies (LEGS) Research Program, La Trobe University, Bundoora, Melbourne 3083, Australia; 2 Health and Rehabilitation Research Institute, AUT University, Auckland, New Zealand; 3 Clinical Services, Walk For Life, Dhaka, Bangladesh; 4 LAMB Project, Rajabashor, Parbatipur, Dinajpur 5250, Bangladesh; 5 Department of Paediatric Orthopaedic Surgery, Royal Aberdeen Childrens Hospital, Aberdeen, UK

## Abstract

**Background:**

‘Walk for Life’ (WFL) is the sustainable clubfoot program in Bangladesh, where there are many challenges in implementing the Ponseti technique in a poor and highly populated country. The relapsing tendency of congenital clubfoot deformity means that initial results may well differ from those of the medium and longer term. Over 10000 children with16668 clubfeet have been treated by WFL since its inception in 2009. Such a large project provides both the need to evaluate each individual child’s case, and also the opportunity to evaluate the wider WFL program results. Such systematic review requires a measure that is sufficiently robust, yet contextually practical, hence the aim of this work was to develop a tool for this purpose, and to report the examiner reliability.

**Methods:**

The Bangla clubfoot tool was largely developed from components of existing validated clubfoot assessment measures, and adapted for local use. Three areas of examination are included: parent satisfaction, gait, clinical examination of the clubfoot. A same-subject repeated measures study design was used to assess the intra-rater reliability of a local WFL physiotherapist, and a visiting WFL volunteer. The inter-rater reliability was also assessed, which is relevant for other examiners and other clubfoot projects undertaking evaluation of medium and longer term results.

**Results:**

The reliability study was conducted in 37 children who had commenced treatment for congenital clubfoot deformity using Ponseti method within the previous two years. The mean age of the children was 2.6 years, with gender 28 male: 9 female. The intra-rater reliability results [ICCs (95% CI)] were: 0.87 (0.76 – 0.93) for the local WFL examiner, and 0.82 (0.64 – 0.91) for the visiting examiner. Inter-rater reliability results [ICCs (95% CI)] were: 0.92 (0.88 – 0.96). Hence the tool showed very good intra-rater and inter-rater reliability, rendering it suitable for use.

**Conclusions:**

The Bangla clubfoot tool has been developed to suit the context of the large WFL clubfoot program in Bangladesh, and shown to be a very reliable evaluation instrument.

## Background

Clubfoot projects are many and varied across the globe, with particular needs and specificities, especially in parts of the developing world. Walk for Life (WFL – http://www.walkforlife.org.au) is the sustainable clubfoot program in Bangladesh, and previous reports have indicated the challenges and successes of implementing the Ponseti technique in this poor and populous country [1]. Whilst many of the conditions faced in Bangladesh are similar to those in other parts of the developing world [2-4], there are also recognised factors that are peculiar to this cultural context and clinical setting viz. a public health system with overcrowded clinics, difficult transport for the lengthy treatment process, poor infrastructure, lack of basic facilities eg hand-washing, adequate lighting [1]. When preparing to evaluate the results of WFL in children two years after treatment commencement, it was recognised that existing assessment tools were not fully commensurate with the WFL database and clinical methods. Hence, a new tool has been developed which is context specific, draws on existing validated instruments where possible, and which will facilitate the ongoing evaluation of the ever increasing WFL case load. Since its inception in 2009, WFL presently (April, 2014) has 16668 feet under correction, in approximately 10000 children (http://www.walkforlife.org.au).

## Methods

Existing clubfoot assessment tools were evaluated for suitability for the Bangladesh context [5-9]. Comprising elements of parent opinion and satisfaction, gait parameters, and clinical foot examination, the new tool is based upon an existing classification model for clubfoot [10] and incorporates facets from a previous long-term evaluation of congenital clubfoot treatment [11]. The resulting *Bangla clubfoot tool* was developed, and evaluated prior to use in auditing clinical outcomes in children at least two years after commencing Ponseti treatment [12] (Tables 1 and 2).

**Table 1:** The Bangla clubfoot tool

					
**A. Parental rating**	**Yes**	**Don’t know**	**No**	**Mean scores (%)**	**‘Rating’**
	**+1**	**0**	**-1**		**#**
1. Happy with child’s feet?					
2. Recommend to others?					
3. Does child play with others?					
4. Does child wear shoes of choice?					
5. Does child have pain?					
*Parental rating sub score (-/5)*					
**B. Gait assessment**	**Yes**	**Not fully/with assistance**	**No**	**Mean scores (%)**	
	**+1**	**0**	**-1**		
6. Squatting					
7. Walking					
8. Running					
9. Up/down steps					
*Gait assessment sub score (-/4)*					
**C. Clinical examination**	**Valgus**	**Straight**	**Varus**	**Mean scores (%)**	
	**+1**	**0**	**-1**		
10. Heel position - L					
Heel position - R					
	**> 0 dorsiflexion**	**0 / 90 degrees**	**< 0 dorsiflexion**	**Mean scores (%)**	
	**+1**	**0**	**-1**		
11. Ankle range - L					
Ankle range - R					
*Clinical examination sub score (-/2)*				L / R	
				^	
Total score (-/11)					

# Note: The clinical examination subscore combines the findings for both heel position in stance and ankle range. Each are ranged from +1 (valgus heel/>0 degrees ankle dorsiflexion) OR 0 (straight heel/0 degrees ankle dorsiflexion) OR -1 (varus heel/<0 degrees ankle dorsiflexion). Neutral to positive scores indicate maintained correction, whilst negative scores are indicative of relapsing deformity.

^ Note: Scores for bilateral cases were halved to achieve same scale/foot for section C/clinical examination.

**Table 2:** The Bangla clubfoot tool score system, which provides easily understood categories

**Total score guide (after Porecha, [**[7]**])**				
Clubfoot	11	9	7	0
‘Grading’	Very good	Good	Fair	Poor
%	85-100	70 - 85	60-70	<50

The guidelines of Bangladesh Medical Research Council (BMRC) and the ethical review committee of World Health Organization (WHO) were used to frame the clinical audit to evaluate the effectiveness of the WFL clubfoot project. The first participants in the clinical audit were simultaneously those assessed for the reliability of the developed assessment tool. All records were collected with permission from the Walk for Life authority. All data anonymity was preserved to maintain patient confidentiality. A request letter was sent to the appropriate authority of the study area to obtain permission for data collection for the clinical audit.

A same subject, repeated measures design was used to evaluate the intra-rater and inter-rater reliability of the new data collection tool in 37 children (who met the study criteria of the subsequent evaluation in 400 children) using two examiners. The examiners were a local Bangladeshi physiotherapist with 15 years experience, and an Australian podiatrist with 25 years experience. The examiners were blinded to each others assessment findings. Each child, was twice assessed by examiners, with not less than two hours separating the examinations (the first assessment forming the clinical audit). This availed the reliability of the local examiner in Bangladesh (intra-rater) who then conducted the subsequent study assessments, bench-marked against the intra-rater reliability of an international examiner. It also provided the inter-rater reliability level for the tool, which is more widely applicable to other examiners and for use in other clubfoot projects [13].

### Participants

The 37 children were of mean age 2.60 years (SD 0.94), and with their parents/carers attended the NITOR hospital outpatients department in central Dhaka for assessment (February, 2013).

Inclusion criteria were that children participating in this study had all been treated for congenital clubfoot deformity using the Ponseti method, within the WFL clinical program. There were no exclusion criteria for the study.

### Measurement

The children’s WFL ID numbers were used to preserve anonymity and to coordinate with the WFL database for the subsequent treatment outcomes audit [12].

The Bangla clubfoot tool comprises three sections of assessment: parent satisfaction, gait function, and clinical foot evaluation (Table 1). The grading system was adopted to facilitate the communication of results, and the percentage basis was used to align with similar tools [7] (Table 2).

### Procedure

The parents of each child were asked to answer five subjective questions: are you happy with your child’s feet; would you recommend this program to others; does your child play with other children; does your child wear shoes of your/their choice; does your child have painful feet. Each child was then observed for aspects of their gait ability: squat, walk, run, up/down steps. The children were both informally observed to walk/run in the clinic space, and also formally seen to walk and run approximately five metres across the clinic (often between the parents). The children were seen to both ascend and descend single steps of approximately 10 cm rise, with either one or both feet placed on each step. Finally, each child had their clubfoot clinically examined to assess: standing heel position, passive ankle range. Heel position was observed posteriorly with the child standing in front of a parent (who would engage the child with a small toy, pen, or ball to achieve static bipedal stance). Ankle dorsiflexion was examined manually with the child sitting supine, with the knee both extended and flexed. The Bangla clubfoot tool can be sub-scored for each of the three domains: parent satisfaction, gait, and clinical; and also summed as a total score which, converted to percentage, indicates the quality/grade of the individual child’s results from treatment (Tables 1 and 2).

### Data management and statistical analysis

After the data collection was completed, all data were entered for reliability analysis using SPSS version 20 (SPSS Inc., Chicago, IL, USA). Intraclass correlations (ICCs) were used for each of the Bangla tool items (ICC (2,k) absolute agreement). Both intra-rater and inter-rater reliability were examined within and between each examiner respectively. The usual interpretation of the returned reliability results was used: values > 0.75 indicate good reliability, values ranging from 0.50 to 0.75 indicate moderate reliability and values < 0.50 imply poor reliability [13].

## Results

Descriptive statistics were used to identify the characteristics of the 37 participating children. The children were of mean age 2.6 years (SD 0.94), with the youngest aged 1.3 years and the oldest 5.0 years. There were 28 boys and 9 girls, as is a typical ratio for clubfoot, which has a male predilection. Typical clubfeet were found in 30 children, with 7 having atypical clubfeet [14]. In 8 cases there was a unilateral left clubfoot, and in 7 cases a unilateral right clubfoot. Both feet were affected in 22 cases. The severity of the clubfeet was indicated by an initial Pirani score average of 4.6 (SD 1.3) left and 4.8 (SD 1.2) right.

Tests of normality were applied using the Shapiro-Wilks statistic. The significance level was >0.5 for the gait and clinical assessment sections, and largely constant for the parental rating (see Tables 3 and 4).

**Table 3:** Intra-rater reliability results for each examiner, across both testing periods (n = 37)

**Variable**	**Trial 1 mean (SD)**	**Trial 2 mean (SD)**	**ICC (95% CI)**
**Rater 1 – local WFL staff**			
1. Happy with child’s feet? mean (SD)	1.00 (0.0)	1.00 (0.0)	1.00 (1.00 – 1.00)
2. Recommend to others? mean (SD)	1.00 (0.0)	1.00 (0.0)	1.00 (1.00 – 1.00)
3. Does child play with others? mean (SD)	1.00 (0.0)	1.00 (0.0)	1.00 (1.00 – 1.00)
4. Does child wear shoes of choice? mean (SD)	1.00 (0.0)	1.00 (0.0)	1.00 (1.00 – 1.00)
5. Does child have pain? mean (SD)	1.00 (0.0)	1.00 (0.0)	1.00 (1.00 – 1.00)
Parental Rating sub score	5.00 (0.0)	5.00 (0.0)	1.00 (1.00 – 1.00)
6. Squatting	0.84 (0.44)	0.81 (0.51)	0.82 (0.66 – 0.91)
7. Walking	0.97 (0.16)	0.97 (0.16)	1.00 (1.00 – 1.00)
8. Running	0.84 (0.55)	0.89 (0.45)	0.53 (0.08 – 0.75)
9. Up/down steps	0.84 (0.37)	0.78 (0.41)	0.79 (0.59 – 0.89)
Gait assessment sub score	3.54 (1.04)	3.43 (1.42)	0.84 (0.71 – 0.92)
10. Heel position - left	- 0.24 (0.62)	-0.31 (0.60)	0.94 (0.88 – 0.97)
Heel position - right	- 0.31 (0.67)	-0.48 (0.62)	0.85 (0.69 – 0.93)
11. Ankle range – 11.Ankle range - left	0.88 (0.32)	0.90 (0.31)	0.52 (-0.04 – 0.77)
Ankle range - right	0.84 (0.36)	0.84 (0.37)	0.75 (0.49 – 0.88)
Clinical examination sub score	0.92 (1.30)	0.81 (1.33)	0.87 (0.76 – 0.93)
Total score	9.01 (2.49)	9.24 (1.91)	**0.87 (0.76 – 0.93)**
**Rater 2 – WFL trainer (visiting)**			
1. Happy with child’s feet? mean (SD)	0.99 (0.47)	1.00 (0.0)	1.00 (0.0)
2. Recommend to others? mean (SD)	1.00 (0.0)	1.00 (0.0)	1.00 (0.0)
3. Does child play with others? mean (SD)	1.00 (0.0)	1.00 (0.0)	1.00 (0.0)
4. Does child wear shoes of choice? mean (SD)	1.00 (0.0)	1.00 (0.0)	1.00 (0.0)
5. Does child have pain? mean (SD)	0.92 (0.36)	0.97 (0.16)	0.48 (0.001 – 0.73)
Parental Rating sub score	4.92 (0.36)	4.97 (0.16)	0.48 (0.001 – 0.73)
6. Squatting	0.84 (0.44)	0.89 (0.39)	0.92 (0.84 – 0.95)
7. Walking	0.97 (0.16)	1.00 (0.0)	1.00 (0.0)
8. Running	0.89 (0.45)	1.00 (0.0)	1.00 (0.0)
9. Up/down steps	0.81 (0.39)	0.86 (0.34)	0.89 (0.79 – 0.94)
Gait assessment sub score	3.51 (1.12)	3.78 (0.47)	0.75 (0.52 – 0.87)
10. Heel position - left	-0.01 (0.56)	-0.03 (0.42)	0.83 (0.64 – 0.92)
- Heel position - right	-0.11 (0.56)	-0.28 (0.52)	0.60 (0.17 – 0.81)
11. Ankle range - left	0.86 (0.34)	0.79 (0.41)	0.69 (0.34 – 0.85)
- Ankle range - right	0.77 (0.42)	0.78 (0.42)	0.77 (0.53 – 0.89)
Clinical examination sub score	1.18 (1.26)	1.05 (0.99)	0.86 (0.73 – 0.93)
Total score	9.10 (2.92)	9.81 (1.17)	**0.82 (0.64 – 0.91)**

**Table 4:** Inter-rater reliability for each measure for each of the repeated trials (n = 37)

**Variable**	**ICC**	**(95% CI)**
1. Happy with child’s feet?	1.00	1.00 – 1.00
mean (SD)		
2. Recommend to others?	1.00	1.00 – 1.00
mean (SD)		
3. Does child play with others?	1.00	1.00 – 1.00
mean (SD)		
4. Does child wear shoes of choice?	1.00	1.00 – 1.00
mean (SD)		
5. Does child have pain?	0.48	0.001 – 0.73
mean (SD)		
Parental Rating sub score	0.48	0.001 – 0.73
6. Squatting	0.93	0.89 – 0.96
7. Walking	1.00	1.00 – 1.00
8. Running	0.78	0.62 – 0.88
9. Up/down steps	0.91	0.85 – 0.95
Gait assessment sub score	0.91	0.86 – 0.95
10. Heel position - left	0.91	0.83 – 0.95
- Heel position - right	0.80	0.64 – 0.89
11. Ankle range - left	0.82	0.68 – 0.91
- Ankle range - right	0.83	0.71 – 0.91
Clinical examination sub score	0.92	0.86 – 0.95
**Total score**	**0.92**	0.88 – 0.96

The intra-rater reliability results for the Bangla tool total score returned ICCs (95% CI) of 0.87 (0.76 – 0.93) for the local WFL examiner, and 0.82 (0.64 – 0.91) for the visiting examiner (Table 3).

Table 4 displays the inter-rater reliability results which returned ICCs (95% CI) of 0.92 (0.88 – 0.96), indicating very good reliability of the assessment tool.

## Discussion

Suitable, repeatable outcome measures are required to determine the results of clubfoot treatment. Most of the clubfoot treatment literature includes only short term results studies which have used measures that may not best represent good and longer term foot function [10]. Dietz et al. evaluation of the Roye instrument supported its use as a clubfoot outcome measure in children by providing evidence of its reliability, validity, and discriminatory power [10].

The Roye tool consists of 10 questions for parents to respond to, and from which the Bangla clubfoot tool parent satisfaction section is derived. The Bangla clubfoot tool then includes clinician observations of children’s gait ability (the Roye tool includes questions of walking, running ability), and also two examination items – ankle range, and standing heel position – which are cardinal signs of both foot function and clubfoot correction.

The Bangla clubfoot tool was developed for the specific purpose of evaluating the results of the WFL clubfoot project in Bangladesh. As a large clubfoot program in a poor and populous country, it is important that such a tool be accurate, expedient, and inexpensive. The return of the reliability study results have demonstrated this simple tool features good reliability both within and between examiners.

Clinicians can feel confident about the use of this tool in the busy clinical setting. The existence of the three domains within the tool also enable partial assessments to be performed, and sub-scored. This tool has been designed to assess results of clubfoot management in children of walking age, ie mid-term to longer-term results. It presupposes walking gait development, which is the ultimate goal of clubfoot treatment. Further evaluation of this tool and the scoring levels needs to explore the validity of both facets, and across age groups. The relationship of the total score with the initial Pirani severity score may shed further light upon outcome predictions.

One of the main concerns at follow up visits for children previously treated for clubfoot deformity is the possibility of relapse [15]. The Bangla clubfoot tool includes the signs (heel position, ankle range) which can indicate relapsing deformity, and also provides for gait observation (where foot supination, or adduction, may be seen).

Whilst not as comprehensive as other clubfoot evaluation tools [10,16], it must be appreciated that the WFL clinics are very busy, commonly seeing 20 or more children with clubfeet in a single clinic session. Thus, the evaluation tool needs to be quick, relevant, and reliable to ensure that it is used and useful. Data arising from assessments will be entered into the WFL national database (as currently occurs with Pirani scores, demographic data, details of the Ponseti treatment course – casts, tenotomy, brace use).

WFL have now innovatively used the Bangla clubfoot tool to report on the findings in 400 children at least two years after their Ponseti management of their clubfeet [12]. The longer term results in these children, and subsequent results in current and future children, necessitate the use of a reliable evaluation that suits the particular and challenging WFL context in Bangladesh. The Bangla clubfoot tool now avails this assessment.

Central to the usefulness of all clinical measures are the two prerequisites of measurement capacity, reliability and validity [13]. A current limitation to the development of this tool is its lack of independent validation against a reference criterion. Whilst comprised of valid component elements [7,10] (rendering face, content validity) and demonstrating good repeatability, the Bangla clubfoot tool should also undergo future evaluation against a ‘gold standard’, and possible refinement.

## Conclusions

The Bangla clubfoot tool has been developed, drawing on existing validated instruments, and reflecting the contextual needs of WFL in Bangladesh. As part of the development The Bangla clubfoot tool has also been examined and shown to be a reliable evaluation instrument. Validation against a criterion will be an important, subsequent investigation.

### Consent

Informed consent was obtained from the patient’s guardian/parent/next of kin for the publication of this report and any accompanying images.

## Competing interests

RP is a WFL physiotherapist, VF-P, SB and AE are WFL volunteers.

## Authors’ contributions

All authors contributed to the study design, data collection, data entry, manuscript review. AE analysed the data and wrote the manuscript draft. All authors read and approved the final manuscript.
